# Quantitative determination of gadolinium and iodine contrast agents in dual-energy computed tomography via a dual-energy iterative reconstruction algorithm: a simulation study on multi-contrast imaging

**DOI:** 10.1093/rpd/ncaf163

**Published:** 2026-03-13

**Authors:** Maria Magnusson, Michael Sandborg, Åsa Carlsson Tedgren, Alexandr Malusek

**Affiliations:** Department of Electrical Engineering, Linköping University, SE-581 83 Linköping, Sweden; Center for Medical Image Science and Visualization (CMIV), Linköping University, SE-581 85 Linköping, Sweden; Center for Medical Image Science and Visualization (CMIV), Linköping University, SE-581 85 Linköping, Sweden; Department of Health, Medicine and Caring Sciences, Linköping University, SE-581 83 Linköping, Sweden; Department of Medical Physics, Linköping University Hospital, SE-581 85 Linköping, Sweden; Center for Medical Image Science and Visualization (CMIV), Linköping University, SE-581 85 Linköping, Sweden; Department of Health, Medicine and Caring Sciences, Linköping University, SE-581 83 Linköping, Sweden; Department of Nuclear Medicine and Medical Physics, Karolinska University Hospital, SE-171 77 Stockholm, Sweden; Center for Medical Image Science and Visualization (CMIV), Linköping University, SE-581 85 Linköping, Sweden; Department of Health, Medicine and Caring Sciences, Linköping University, SE-581 83 Linköping, Sweden

## Abstract

Simultaneous quantification of two contrast agents in dual-energy CT (DECT) is promising for clinical diagnostics. This simulation study demonstrates that accurate estimation of iodine and gadolinium concentrations in unmixed aqueous solutions can be achieved using two-material decomposition and beam-hardening suppression with the dual-energy iterative reconstruction algorithm (DIRA). A simulated DECT imaging chain was used for evaluation. The phantom comprised a lipid cylinder containing five inserts filled with water, iodine (25 and 50 mg/mL), and gadolinium (25 and 50 mg/mL) solutions. X-ray projections at 80 and Sn140 kV were generated using Siemens’ DRASIM code. DIRA was configured for two-material decomposition with water–bone, water–iodine, and water–gadolinium bases, and cylinder positions were manually defined during reconstruction. After approximately six iterations, accurate estimates of contrast agent mass fractions were obtained. These results confirm the feasibility of simultaneous iodine and gadolinium quantification in DECT and support further development toward a clinical application.

## Introduction

The exploration of alternative contrast media with distinct K-edge energies compared to iodine has emerged as a promising strategy for advancing diagnostic imaging [1, 2]. The ability to exploit the K-edge of various materials provides a powerful tool for imaging and materials analysis, enabling a detailed assessment of chemical composition, electronic structure, and the local atomic environment of the absorbing element [3]. Using multiple contrast agents in clinical imaging offers potential benefits such as improved diagnostic accuracy through better differentiation of uptake and distribution patterns [4]. Moreover, this approach can streamline the workflow and reduce repeated scans, as contrast separation can be achieved through post-processing from a single acquisition. Currently, iodine-based agents are the most widely used and approved for X-ray imaging, whereas gadolinium-based agents are primarily used in magnetic resonance imaging (MRI). Nevertheless, gadolinium can also serve as a contrast medium in X-ray imaging, although higher concentrations are required for sufficient detectability [5].

In computed tomography (CT), projection data (also called raw data or sinograms) are measured by the detector and reconstructed into images of linear attenuation coefficients (LACs). The filtered backprojection (FBP) is a conventional analytical reconstruction algorithm that produces voxel values representing the X-ray attenuation properties of different materials. These are typically presented as CT numbers defined as $H = 1000 \cdot \left ( {\mu }/{\mu _{w}} - 1 \right )$, where $\mu$ and $\mu _{w}$ denote the LACs of the material and water, respectively, at a specific photon energy. As a result, the CT number of water is 0 Hounsfield Units (HU). In this work, we use LACs—the primary physical quantities reconstructed from projection data—rather than the derived CT numbers.

**Figure 1:**
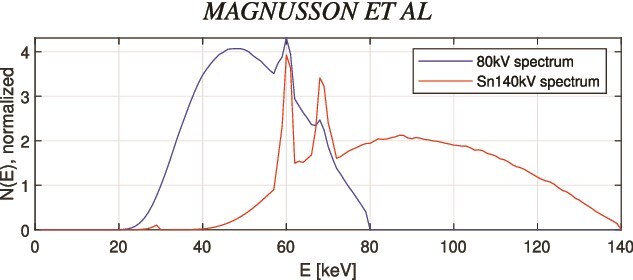
Normalized photon energy spectra for the two X-ray tube settings used in the simulations: 80 kV (left curve) and Sn140 kV (right curve). The high-energy spectrum was filtered with tin (Sn) to increase spectral separation.

**Figure 2:**
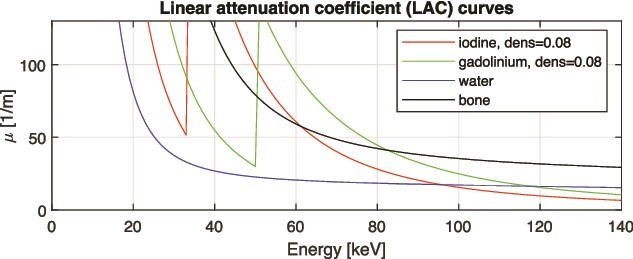
Energy dependence of LACs for iodine, gadolinium, water, and bone. The discontinuities at 33.2 and 50.2 keV correspond to the K-edges of iodine and gadolinium, respectively.

The X-ray tube emits photons with a broad energy spectrum (see Fig. 1), and scanned objects usually contain multiple materials. The LACs exhibit strong energy dependence (Fig. 2). The human body mainly consists of elements with atomic numbers between 1 and 20—such as hydrogen, carbon, nitrogen, oxygen, and calcium—with trace amounts of heavier elements. For such elements, LACs decrease smoothly with increasing energy in the diagnostic energy range of 20–150 keV, as illustrated by water and bone in Fig. 2. In contrast, common contrast agents such as iodine and gadolinium exhibit abrupt increases at their K-edges (33.2 and 50.2 keV, respectively).

The strong energy dependence of LACs and the broad X-ray spectrum cause reconstructed LACs to represent spectrum-averaged values. Classical single-energy CT reconstruction algorithms, such as filtered backprojection, assume a monoenergetic beam and therefore produce beam-hardening artifacts, which are usually reduced—though not eliminated—by water-based correction methods.

Dual-energy computed tomography (DECT), which acquires two datasets using different tube voltages, can more effectively suppress beam-hardening artifacts, provide more accurate LAC and CT numbers, and improve material characterization [6].

DECT reconstruction algorithms are generally classified into projection-based basis material decomposition (PBBMD) and image-based basis material decomposition (IBBMD) methods [6]. PBBMD techniques—such as the widely used Alvarez–Macovski method [7]—require geometrically consistent projection data and are limited to representing the entire object with a single pair of basis materials, which are typically selected by the user before reconstruction. In contrast, IBBMD methods offer greater flexibility by allowing different material bases, such as tissue-specific doublets or triplets, to be applied to different organs or regions within the same image. However, because IBBMD operates in the image domain, it remains more susceptible to beam-hardening artifacts.

A hybrid approach is the multi-material decomposition method implemented in General Electric’s fast kVp-switching DECT scanners. In this method [8], projection-based dual-energy data are first reconstructed into quantitative basis-material density images, typically representing water and iodine. Building on these, a three-material decomposition is performed to estimate material fractions under volume-preserving conditions. The framework is further generalized through a hierarchical library of material triplets that tessellate the attenuation space, enabling robust and physically consistent decomposition across multiple materials.

A clinical implementation of the IBBMD approach is the Siemens’ proprietary Monoenergetic Plus algorithm [9] for DECT, which we evaluated in a previous study [10]. The algorithm performed well overall; however, minor discrepancies in CT numbers and residual beam-hardening artifacts were observed. Beam-hardening artifacts were also observed by Wohlfahrt *et al*. [11].

A method that simultaneously operates on both projection and image data is the Dual-Energy Iterative Reconstruction Algorithm (DIRA) [12], capable of processing inconsistent rays from dual-source DECT scanners. The original DIRA algorithm is 2D, iterative, and incorporates FBP within each iteration. It supports both two- and three-material decomposition schemes to model body tissues. The 3D extension, DIRA-3D, accommodates dual-energy, dual-source helical CT data [13]. Simulation studies have shown that both DIRA and DIRA-3D can produce CT numbers that are quantitatively accurate.

This simulation study aims to demonstrate that two-material decomposition within DIRA can (i) obtain CT numbers of iodine and gadolinium solutions close to tabulated values from the EPDL97 library [14] and (ii) accurately determine the concentrations of iodine and gadolinium contrast agents within the same phantom, where both agents are present simultaneously but spatially separated.

## Materials and methods

### Simulation framework

A DECT imaging chain was simulated to evaluate the proposed method. Photon energy spectra corresponding to X-ray tube voltages of 80 and 140 kV were employed (Fig. 1). The high-energy spectrum was additionally filtered with a tin (Sn) filter and is therefore denoted Sn140 kV. X-ray projections for both spectra were generated using DRASIM, a CT simulation tool developed by Siemens [15]. Image reconstruction was performed using the DIRA code, adapted for the quantification of contrast agents.

### Phantom

**Figure 3:**
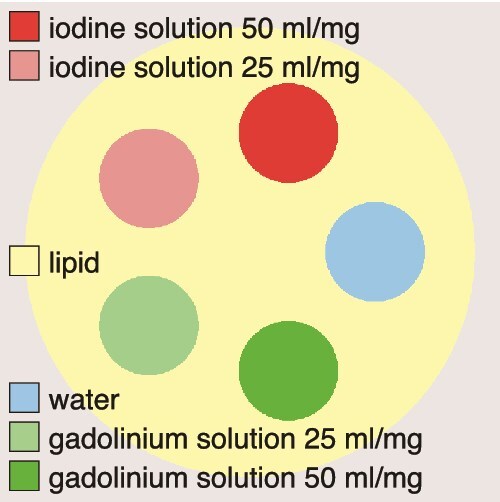
Schematic illustration of the cylindrical lipid phantom used in the simulations. The phantom (diameter = 31.6 cm) contained five 7 cm-diameter inserts filled with water, iodine solutions (25 and 50 mg/mL), and gadolinium solutions (25 and 50 mg/mL).

The imaged object (Fig. 3) was a torso-sized cylindrical lipid phantom with a diameter of 31.6 cm. It contained five internal cylinders, each 7 cm in diameter, filled with either water, iodine solutions (25 mg/mL and 50 mg/mL), or gadolinium solutions (25 and 50 mg/mL).

Figure 2 shows the LACs as a function of photon energy for iodine, gadolinium, water, and bone. In the diagnostic CT energy range (30–150 keV), the LACs of water and bone decrease monotonically with energy, as is also the case for lipid and other biological tissues composed primarily of elements with atomic numbers $Z \leq 20$. In contrast, iodine and gadolinium exhibit characteristic discontinuities at their K-edges (33.2 and 50.2 keV, respectively).

### Two-material decomposition

Two-material decomposition models each voxel as a mixture of two predefined base materials. For each voxel, the method estimates the mass fractions of the two materials, $w_{1}$ and $w_{2} = 1 - w_{1}$, together with the total mass density $\rho$ of the mixture.

In this study, the decomposition scheme was adapted to the type of tissue or contrast agent present in the phantom. Soft tissue regions (represented by lipid and water) were decomposed using a water–bone basis, whereas the iodine and gadolinium solutions were decomposed using the iodine–water and gadolinium–water bases, respectively.

### Effective energies in DIRA

In DIRA, the effective energy of each X-ray spectrum was determined from the corresponding attenuation behavior of water. First, the effective linear attenuation coefficient, $\mu _{\mathrm{eff}}$, was computed as the spectrum-weighted mean of the water attenuation curve $\mu (E)$. The energy value on the $\mu (E)$-curve that corresponded to $\mu _{\mathrm{eff}}$ was then defined as the effective energy. Two effective energies, $E_{\mathrm{eff,L}}$ and $E_{\mathrm{eff,H}}$, representing the low- and high-energy spectra, respectively, were selected. For the 80 and Sn140 kV spectra (Fig. 1), the effective energies were approximately 50 and 88 keV. To avoid numerical instability near the gadolinium K-edge (50.2 keV), a slightly higher value of 55 keV was used in the simulations.

### DIRA algorithm workflow

**Figure 4:**
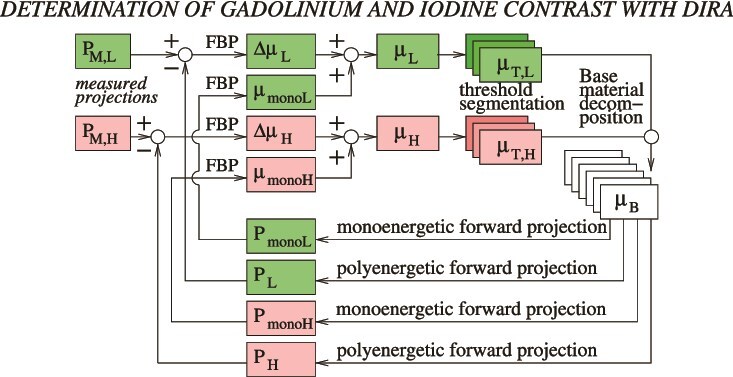
Flowchart of the dual-energy iterative reconstruction algorithm DIRA adapted for contrast agent quantification.

The DIRA algorithm, adapted for contrast quantification in this study, is outlined in Fig. 4 and summarized below.

1. *Acquisition.* Measured projections $P_{\mathrm{M,L}}$ and $P_{\mathrm{M,H}}$ are obtained for the low (80 kV) and high (Sn140 kV) energy spectra, respectively.
2. *Initial reconstruction.* The projections are reconstructed using FBP to LAC images $\mu _{\mathrm{L}}$ and $\mu _{\mathrm{H}}$. The first iteration (iteration 0) includes a conventional water beam-hardening correction (not shown in the figure).
3. *Segmentation.* Regions corresponding to (a) the soft tissue, (b) the iodine solution, and (c) the gadolinium solution are segmented, yielding six images $\mu _{\mathrm{T,L}}$ and $\mu _{\mathrm{T,H}}$ in total ($2$ energies $\times$  $3$ materials).
4. *Two-material decomposition.* Each segmented region is decomposed using appropriate material pairs: (a) water–bone, (b) iodine–water, and (c) gadolinium–water. The algorithm estimates the mass fraction ($w_{1}$) and total density ($\rho$) for each basis pair. Because $w_{2} = 1 - w_{1}$, only one mass-fraction image per basis is stored, resulting in six images in total (three bases $\times$ two outputs: $w_{1}$ and $\rho$).
5. *Monoenergetic forward projection.* Forward projections $P_{\mathrm{mono,L}}$ and $P_{\mathrm{mono,H}}$ are generated for 55 keV and 88 keV, close to the effective energies $E_{\mathrm{eff,L}}=50~\mathrm{keV}$ and $E_{\mathrm{eff,H}}=88~\mathrm{keV}$, respectively. These are reconstructed to monoenergetic LAC images $\mu _{\mathrm{mono,L}}$ and $\mu _{\mathrm{mono,H}}$.
6. *Polyenergetic forward projection and correction.* Polyenergetic forward projections $P_{\mathrm{L}}$ and $P_{\mathrm{H}}$ are simulated and compared with the measured projections. The resulting residuals are reconstructed by FBP to obtain correction terms $\Delta \mu _{\mathrm{L}}$ and $\Delta \mu _{\mathrm{H}}$, which are added to the monoenergetic images. This produces updated $\mu _{\mathrm{L}}$ and $\mu _{\mathrm{H}}$ for the next iteration.

Steps 2–6 are iterated a predefined number of times (6 and 16 iterations were used in this study). After the final iteration, approximately monoenergetic images at 55 and 88 keV are obtained, corresponding to $\mu _{\mathrm{L}}$ and $\mu _{\mathrm{H}}$, respectively.

### Data analysis

**Figure 5:**
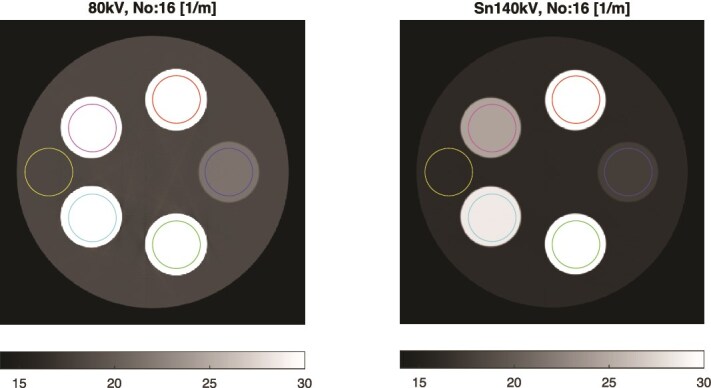
DIRA reconstructions of LACs ($\mathrm{m}^{-1}$) after 16 iterations at effective energies of 55 keV (left) and 88 keV (right). Mean LACs were measured within the colored circular regions.

For presentation and quantitative evaluation, mean values were calculated within the colored circular regions shown in Fig. 5. Each value is reported as the mean $\pm$ standard deviation of all values corresponding to the pixels inside the specified region. Variations in the reconstructed LACs arose from reconstruction imperfections; quantum noise in the projections was not simulated.

## Results

**Figure 6:**
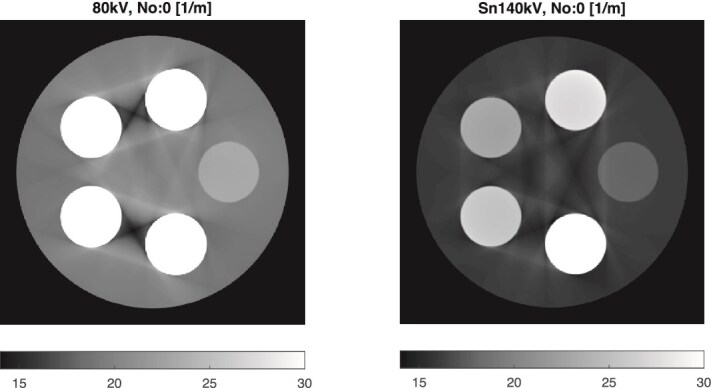
DIRA reconstructions of LACs ($\mathrm{m}^{-1}$) after iteration 0 (equivalent to FBP). Left: 80 kV; right: Sn140 kV. Pronounced beam-hardening artifacts appear as dark regions between the iodine and gadolinium inserts.

Images of the phantom were reconstructed using the DIRA algorithm. Figure 6 shows the reconstruction after iteration 0, where pronounced beam-hardening artifacts appear as dark regions between the iodine and gadolinium rods. After 16 iterations (Fig. 5), these artifacts were effectively removed, yielding a more uniform image representation.

The reconstructed LACs, $\mu _{L}$ and $\mu _{H}$, are summarized in Table 1. Compared with reference values derived from EPDL97 data [14], the results show rapid convergence of DIRA with iteration. By iteration 6, the reconstructed LACs agreed with the references within approximately 2%, and by iteration 16, within about 1%. For water and lipid, the deviations were already small—less than about 7% at iteration 0 and 3% at iteration 1—while the LAC for 50 mg/mL gadolinium was initially underestimated by roughly 32%. These results confirm that DIRA effectively compensates for beam-hardening effects and yields quantitatively accurate LACs after a limited number of iterations.

**Table 1:** Reconstructed linear attenuation coefficients (m$^{-1}$) at different DIRA iterations for the various materials, compared with reference values from the EPDL97 library. Values are mean $\pm$ SD within circular regions of radius 40 pixels. Standard deviations smaller than 0.05 are shown as 0.0 due to rounding.

Material	True LACs at **55** keV	LACs at iter 0	LACs at iter 1	LACs at iter 6	LACs at iter 16
50 mg/mL I	**68.5**	58.4 $\pm$ 1.0	66.3 $\pm$ 0.8	68.9 $\pm$ 0.1	68.8 $\pm$ 0.0
25 mg/mL I	**45.1**	42.4 $\pm$ 0.6	45.8 $\pm$ 0.4	45.4 $\pm$ 0.1	45.3 $\pm$ 0.0
50 mg/mL Gd	**94.4**	64.6 $\pm$ 0.9	76.3 $\pm$ 1.0	92.4 $\pm$ 0.4	94.1 $\pm$ 0.1
25 mg/mL Gd	**58.0**	45.2 $\pm$ 0.4	51.0 $\pm$ 0.4	57.5 $\pm$ 0.1	57.9 $\pm$ 0.1
Water	**21.5**	23.1 $\pm$ 0.1	22.0 $\pm$ 0.1	21.5 $\pm$ 0.1	21.5 $\pm$ 0.1
Lipid	**18.4**	19.0 $\pm$ 0.2	17.9 $\pm$ 0.1	18.4 $\pm$ 0.1	18.4 $\pm$ 0.1
**Material**	**True LACs at 88 keV**	**LACs at iter 0**	**LACs at iter 1**	**LACs at iter 6**	**LACs at iter 16**
50 mg/mL I	**31.1**	27.4 $\pm$ 0.3	30.2 $\pm$ 0.2	31.2 $\pm$ 0.0	31.2 $\pm$ 0.0
25 mg/mL I	**24.4**	22.8 $\pm$ 0.1	24.4 $\pm$ 0.1	24.5 $\pm$ 0.0	24.5 $\pm$ 0.0
50 mg/mL Gd	**39.2**	32.6 $\pm$ 0.4	35.7 $\pm$ 0.3	38.9 $\pm$ 0.1	39.3 $\pm$ 0.0
25 mg/mL Gd	**28.5**	25.6 $\pm$ 0.2	27.2 $\pm$ 0.1	28.5 $\pm$ 0.0	28.6 $\pm$ 0.0
Water	**17.8**	17.9 $\pm$ 0.1	17.9 $\pm$ 0.0	17.8 $\pm$ 0.0	17.8 $\pm$ 0.0
Lipid	**16.1**	16.1 $\pm$ 0.2	16.0 $\pm$ 0.1	16.1 $\pm$ 0.0	16.1 $\pm$ 0.0

The reconstructed mass fractions, summarized in Table 2, exhibit similar convergence behavior. At iteration 0, the high iodine mass fraction was underestimated by about 12%, but this deviation decreased to within 1% of the reference by iteration 6. For gadolinium, the initial estimates were substantially lower—43%–53% of the true values—but improved rapidly, reaching agreement within 4% at iteration 6 and 1% at iteration 16.

**Table 2:** Reconstructed iodine and gadolinium mass fractions at successive DIRA iterations for the considered solutions. Mean $\pm$ SD values are the mass-fraction $w_{1}$ images within circular regions of radius 40 pixels.

Solution	True	Iteration 0	Iteration 1	Iteration 6	Iteration 16
50 mg/mL I	**4.76%**	4.20% $\pm$ 0.15%	4.66% $\pm$ 0.13%	4.80% $\pm$ 0.01%	4.79% $\pm$ 0.01%
25 mg/mL I	**2.44%**	2.51% $\pm$ 0.10%	2.62% $\pm$ 0.07%	2.47% $\pm$ 0.01%	2.46% $\pm$ 0.01%
50 mg/mL Gd	**4.76%**	2.23% $\pm$ 0.09%	2.98% $\pm$ 0.09%	4.55% $\pm$ 0.05%	4.73% $\pm$ 0.02%
25 mg/mL Gd	**2.44%**	1.40% $\pm$ 0.04%	1.78% $\pm$ 0.03%	2.39% $\pm$ 0.02%	2.43% $\pm$ 0.01%

**Figure 7:**
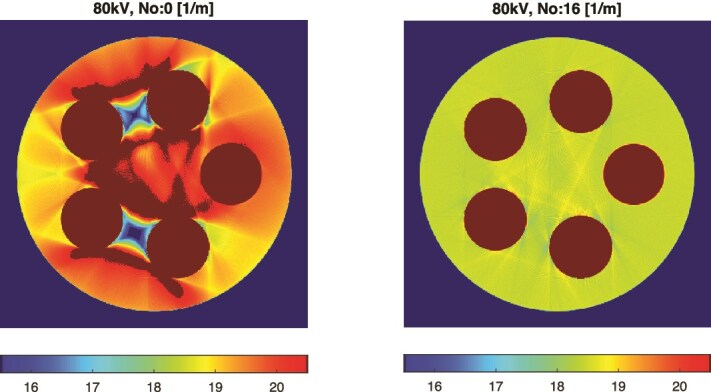
Reduction of beam-hardening artifacts from iteration 0 (left) to iteration 16 (right). The lipid region (true $\mu = \mathrm{18.4~m}^{-1}$ at 55 keV) becomes uniform after the iterative correction.

Figure 7 illustrates the progressive reduction of beam-hardening artifacts. Initially, the presence of iodine and gadolinium produced strong distortions in the surrounding lipid regions. At 55 keV, the true lipid LAC is 18.4 m$^{-1}$, corresponding to the green-yellow hue in the visualization. After 16 iterations, the distortions were largely eliminated, demonstrating that DIRA successfully restores both an artifact-free image appearance and quantitative accuracy.

## Discussion and future work

This simulation study demonstrates that the DIRA algorithm, using region-specific two-material decomposition, enables accurate quantification of iodine and gadolinium contrast agents from DECT data. The estimated LACs and mass fractions converged towards EPDL97-based reference values after approximately six iterations, accompanied by a concurrent suppression of beam-hardening artifacts (Tables 1, 2, and Figs 5– 7).

The use of water–bone as the default tissue pair is supported by previous studies [16, 17], where the water-iodine base gave LAC errors for bone of approximately 2.5% at 50 keV and visible beam-hardening artifacts. This relatively small error likely arises because the iodine K-edge (33.2 keV) is near the lower end of the 80 kV spectrum (Fig. 1).

In contrast, the gadolinium K-edge (50.2 keV) lies near the middle of the 80 kV spectrum, which prevents a water–iodine base from accurately approximating the LAC of gadolinium-containing materials across the diagnostic energy range. This limitation was confirmed by Baubeta *et al*. [18], who reported that no gadolinium K-edge signal could be detected using Siemens DECT or photon-counting CT systems equipped with the SyngoVia software. Conversely, a water–gadolinium base cannot accurately represent the attenuation behavior of human tissues. These observations highlight the importance of region-specific base material selection for accurate gadolinium quantification.

Compared with adaptive model-based material decomposition methods, such as those proposed by Mendonça *et al*. [8], which assume a fixed base pair (typically water–iodine) for the initial PBBMD, the DIRA framework may enhance the result by iteratively (i) adapting base materials to various tissue types within the image domain, and (ii) incorporating their influence into the projection data [16, 17]. In the future, a three-material decomposition of blood vessels in the (water, iodine, gadolinium) basis, performed in the image domain, will influence the forward projections and ultimately converge with the measured projections.

Furthermore, unlike commercial IBBMD methods such as Siemens’ Monoenergetic Plus [9], DIRA integrates projection- and image-domain information, resulting in more efficient beam-hardening correction [10]. The Siemens’ implementation of Monoenergetic Plus assumes a single two-material base, typically water–iodine or water–bone, which limits its applicability for gadolinium as mentioned above.

This study has several limitations. First, it is a simulation study, and no experimental data were analyzed. Second, for simplicity, the regions containing the materials of interest were manually defined in the material decomposition routine. Automating this step using threshold-based or machine learning–based segmentation methods will be an important next step. Third, the assumption of a two-material composition per voxel becomes invalid when contrast agents mix or spatially overlap, motivating future extensions of DIRA to three-material decomposition with water, iodine, and gadolinium as the base material triplet. Fourth, quantum noise in the projections was not simulated. Although including noise would increase the standard deviations reported in Tables 1 and 2, our previous study [12] suggests that realistic noise levels would only have minor effects on the mean values. Future work should also explore the integration of DIRA with photon-counting CT, which may further improve quantitative accuracy and material characterization through enhanced spectral resolution and energy discrimination.

In summary, the results demonstrate the potential of DIRA for accurate dual-contrast quantification in DECT and motivate further validation with experimental and clinical data.

## Conclusion

This simulation study demonstrates that the DIRA algorithm, using region-specific two-material decomposition, can accurately recover the LACs of iodine and gadolinium solutions, yielding values close to those tabulated in the EPDL97 library. The iterative reconstruction process progressively refined the LAC estimates, enabling the precise determination of iodine and gadolinium mass fractions within the same phantom, where both contrast agents were present simultaneously but spatially separated. These results highlight the method’s potential for accurate dual-contrast quantification in DECT and support its further evaluation with experimental and clinical data.
